# Retraction Note: Fire and summer temperatures work together breaking physical seed dormancy

**DOI:** 10.1038/s41598-021-93139-2

**Published:** 2021-06-28

**Authors:** Belén Luna

**Affiliations:** Departamento de Ciencias Ambientales, Facultad de Ciencias Ambientales y Bioquímica, Universidad de Castilla-La Mancha, 45071 Toledo, España

Retraction of: *Scientific Reports* 10.1038/s41598-020-62909-9, published online 07 April 2020

The Editors have retracted this Article

After publication, concerns were raised with respect to whether the experiments had been appropriately controlled. Post-publication peer review confirmed that a synergistic effect is not supported by the data presented in Figure 3, as this experiment lacked a control for the isolated effect of summer temperature. In addition, for Figure 1 and 2, the time at which heat-shock was applied differed between treatments, and they are therefore not comparable.

Belen Luna does not agree to this retraction.

